# Ethnical Variations in the Incidence of Congenital Heart Defects in Gorgan, Northern Iran: A Single-Center Study

**Published:** 2014-01-12

**Authors:** Bagher Nikyar, Maliheh Sedehi, Mostafa Qorbani, Arash Nikyar, Mohammad Jafar Golalipour

**Affiliations:** 1 *Gorgan Congenital Malformations Research Center, Golestan University of Medical Sciences, Gorgan, Iran.*; 2 *Department of Public Health, Alborz University of Medical Sciences, Karaj, Iran.*; 3 *Non-Communicable Diseases Research Center, Endocrinology and Metabolism Research Institute, Tehran University of Medical Sciences, Tehran, Iran.*; 4 *Cranfield University, London, England. *

**Keywords:** Heart defect, congenital • Heart septal defect, atrial • Heart septal defect, ventricular • Ethnic group • Incidence

## Abstract

***Background*** *: *Congenital heart disease (CHD) is the most common congenital anomaly in newborns. This study was performed to determine the live birth incidence of CHD by ethnicity and sex in Gorgan, Northern Iran.

***Methods: ***In this longitudinal, hospital-based study, 18162 live births in Dezyani Hospital in Gorgan, North of Iran, were screened for CHD, from 2007 through 2009. Clinical examination, echocardiography, color Doppler, and cardio catheterization were used as diagnostic tools. Sex, ethnicity, and type of CHD for each case were recorded in a pre-designed questionnaire.

***Results*** *: *The incidence rates of CHD in the native Fars, Sistani, and Turkmen subjects were 5.73 (95%CI: 4.53-7.15), 12.27 (95%CI: 8.74-16.73), and 15.93 (95%CI: 10.00-24.02) per 1000 live births, respectively. The Turkmen to native Fars and Sistani to native Fars relative risk for congenital CHD malformations was 2.77 (95%CI: 1.73-4.44; p value < 0.001) and 1.29 (95%CI: 0.77-2.18; p value < 0.323), respectively. While atrial septal defect was the most common lesion in the native Fars subjects (2.14 per 1000 [95%CI: 1.42-3.06]) and in the Sistani subjects (2.84 per 1000 [95%CI: 1.29-5.36]), in the Turkmen subjects, ventricular septal defect (4.36 per 1000 [95%CI: 1.59-9.43]), followed by atrial septal defect, was the most frequent lesion.

***Conclusion*** *: *This study showed that the incidence and pattern of CHD among live births in Gorgan, North of Iran, varied according to ethnicity. The risk of CHD was higher in the Turkmen and Sistani groups than in the Fars population.

## Introduction

Congenital heart disease (CHD) is the most common and serious congenital anomaly in newborns with a major impact on infant morbidity, mortality, and health care costs.^1^^-^^4^ Determining the prevalence and pattern of CHD can help pediatricians and public health professionals better assess health care needs, evaluate potential clusters, and recommend valuable changes in health policies (Vaidyanathan B, Krishna kumar R. The global burden of congenital heart disease. Congenital Cardiology Today 2005;3:1-8.). CHD has annual rates of 4 to 50 per 1000 live births and is as such an extremely prevalent defect.^5^ Several studies in Iran have reported CHD prevalence rates of between 0.92-8.6 per 1000.^6^^, ^^7^

CHD is a multifactorial disease. Indeed, CHD can be allied to defects in chromosomes or genes as well as environmental factors.^8^ Racial/ethnic differences have been previously highlighted in relation to the prevalence of CHD.^9^^-^^14^ Differences in genetic predisposition, environmental exposures, access to care (diagnosis of CHD), and response to physiologically-based CHD interventions have been cited as probable reasons behind the role of racial/ethnic differences in the prevalence of CHD.^8^^, ^^15^^, ^^16^ Also believed to be influential in CHD prevalence are sex differences.^17^^, ^^18^ Be that as it may, there is currently a paucity of data on differences in CHD rates with respect to maternal race/ethnicity and infant’s sex.^8^^, ^^19^^, ^^20^

There are three major ethnic groups, namely native Fars, Turkmen, and Sistani, in northern Iran. Given the dearth of information on the incidence and pattern of CHD vis-à-vis ethnicity, we sought to determine the live birth prevalence of CHD by race/ethnicity-sex in the northern Iranian city of Gorgan.

## Methods

This longitudinal and hospital-based investigation was undertaken on 18162 live births to identify all newborns with congenital heart malformations born between January 1, 2007 and December 31, 2009, in Dezyani (a teaching and referral hospital which is the main site for about 80% of deliveries in Gorgan, the capital city of the Golestan Province in the north of Iran). This hospital has an annual rate of more than 6000 deliveries, accounting for 20% of all annual births in the Golestan Province and the largest portion of deliveries (80%) in the city of Gorgan itself. The Golestan Province has a population of about 1.8 million and covers an area of about 20460 km^2^. 

The study was approved by the Ethic Committee of Golestan University of Medical Sciences, and parental consent was obtained from the parents of all the subjects along with a clearance from the institutional Ethics Committee.

The patients were primarily from moderate to low socioeconomic class families. All live newborns delivered in this hospital during the investigation were examined and screened for CHD. Each live newborn was visited by a neonatologist. Newborns with cardiac symptoms were referred to the Heart Clinic, where they were managed by pediatric cardiologists. Clinical examination, two-dimensional echocardiography, color Doppler, and cardiac catheterization were implemented as the definitive tools for the diagnosis of CHD.

Different types of CHD considered for the present investigation were comprised of ventricular septal defect (VSD), atrial septal defect (ASD), Tetralogy of Fallot, patent ductus arteriosus (PDA), pulmonary stenosis, transposition of the great arteries, total anomalous pulmonary venous connection, partial anomalous pulmonary venous connection, pulmonary atresia, single ventricle, Ebstein’s anomaly, and complex CHDs (various types of CHDs existing together, including rare types of CHDs). The recorded variables included the date of birth, sex, type of malformations, ethnicity of parents and the presence of other congenital malformations. The total number of live figures of every year was recorded. According to their ethnicity, three ethnic groups (native Fars, Turkmen, and Sistani) residing in this region were considered. The native Fars group is the predominant inhabitant of the region, the Turkmen are those who emigrated from the central Asia more than three centuries ago, and the Sistani people emigrated from the Iran-Pakistan-Afghanistan border (Sistan and Baluchestan Province in the South-East of Iran) more than half a century ago. Ethnicity was determined based on maternal self-report.

***Statistical Analysis***

The descriptive data are presented as percentages. The descriptive statistics were calculated for CHD prevalence per 1000 live births. The prevalence of CHD was calculated as follows: annual rate = CHD cases / total live births. Confidence interval (95%CI) for prevalence was calculated using binomial exact methods. STATA8/SE statistical package was employed for statistical analysis. A p value < 0.05 was considered significant.

## Results

The incidence rates of CHD were 5.73 (95%CI: 4.53-7.15 [male = 6.41 and female = 5.03]), 12.27 (95%CI: 8.74-16.73 [male = 13.12 and female = 11.40]), and 15.93 (95%CI: 10.00-24.02 [male = 15.80 and female = 16.05]) per 1000 live births in the native Fars, Sistani, and Turkmen groups, respectively (Table 1).

**Table 1:** Rate of congenital heart disease per 1000 according to ethnicity and sex in northern Iran (2007-2009)

Ethnicity	Total	Male	Female
Native Fars	5.73	6.41	5.03
Turkmen	15.93	15.80	16.05
Sistani	12.27	13.12	11.40

The male-to-female relative risk for CHD was 1.17 (95%CI: 0.84-1.63; p value = 0.343). The Turkmen to native Fars and Sistani to native Fars relative risk for CHD was 2.77 (95%CI: 1.73-4.44; p value < 0.001) and 1.29 (95%CI: 0.77-2.18 p value = 0.323), respectively (Table 2).

The pattern and number of CHD according to gender and ethnicity are depicted in Table 3. 

In the native Fars ethnic group, ASD was the most common lesion (2.14 per 1000, 95%CI: 1.42-3.06), followed by ASD with VSD (0.73 per 1000, 95%CI: 0.35-1.35) and VSD and PDA (0.44 per 1000, 95%CI: 0.16-0.95), respectively. The rate of ASD in the males and females was 2.92 and [1.33] per 1000, respectively (Table 4). 

In the Sistani ethnic group, ASD was the most common lesion (2.84 per 1000, 95%CI: 1.29-5.36), followed by VSD and PDA (1.89 per 1000, 95%CI: 0.69-4.10). The rate of ASD with VSD in the males and females was 1.25 and 0.63 per 1000, respectively (Table 4). 

In the Turkmen ethnic group, VSD with ASD was the most frequent lesion (4.36 per 1000, 95%CI: 1.59-9.43), followed by ASD (3.63 per 1000, 95% CI: 1.17-8.42), VSD (2.90 per 1000, 95%CI: 0.79-7.40), and PDA (2.17 per1000, 95%CI: 0.44-6.33). The rate of VSD with ASD in the males and females was 2.88 and 5.87 per 1000, respectively (Table 4). 

The rates of ASD, ASD with VSD, VSD, and PDA in the male and female infants were 2.72 and 2.01 per 1000, 0.76 and 1.34 per 1000, 1.30 and 0.33 per 1000, and 0.65 and 1.01 per 1000, respectively (Table 4).

**Table 2:** Association between sex and ethnicity with congenital malformations incidence

		Live Newborns	Newborns with Congenital Malformations	%	Relative risk	95% CI	P value
Sex							
Female		8957	63	0.70	1	-	
Male		9205	76	0.82	1.17	0.84-1.63	0.343
						
Ethnicity							
Fars		13603	78	0.57	1	-	
Turkmen		1381	22	1.59	2.77	1.73-4.44	< 0.001
Sistani		3178	39	1.22	1.29	0.77-2.18	0.322

**Table 3:** Cardiovascular malformations distribution by ethnicity and sex

	Ethnicity								Ethnicity					
	Native Fars		Turkmen		Sistani				Native Fars		Turkmen		Sistani	
	Male	female	Male	female	Male	female			Male	female	Male	female	Male	female
ASD	20	9	2	3	3	6		VSD & ASD & PDA	3	1	0	0	0	0
VSD	3	2	4	0	5	1		PS & TR & VSD	0	0	0	0	2	0
PDA	3	3	0	3	3	3		MVP	1	0	0	0	0	0
ASD & VSD	3	7	2	4	2	1		COA & PDA & PH	0	1	0	0	0	0
TOF	0	1	0	0	1	0		TR & ASD	1	3	0	0	0	1
MR	1	0	1	0	0	0		TR & PDA	0	0	0	0	0	1
ASD & PDA	0	1	0	0	1	1		PE	0	0	0	0	0	1
VSD & PDA	0	0	0	0	1	1		VSD & PS	1	0	0	0	0	0
TR	2	1	0	1	0	0		PR	1	1	0	0	0	0
PH	1	0	0	0	0	0		PDA & TGA	0	0	0	0	0	1
PS	1	2	0	0	1	0		EA	1	0	0	0	0	0
PFO	1	0	0	0	0	0		ASD & TGA	1	0	0	0	0	1
TR & PR	0	0	1	0	2	0		PS & ASD	0	1	0	0	0	0
PR & PS	0	0	1	0	0	0		TGA	0	1	0	0	0	0

ASD, Atrial septal defect; VSD, Ventricular septal defect; PDA, Patent ductus arteriosus; TOF, Tetralogy of Fallot; MR, Mitral regurgitation; TR, Tricuspid regurgitation; PH, Pulmonary hypertension; PS, Pulmonary stenosis; PFO, Patent foramen ovale; PR, Pulmonary regurgitation; MVP, Mitral valve prolepse; COA, Coarctation of the aorta; PE, Pericardial effusion; TGA ,Transposition of great arteries; EA, Ebstein anomaly

**Table 4:** Rate of selected congenital heart disease per 1000 according to ethnicity and gender

Ethnicity	ASD		ASD & VSD		VSD		PDA	
	Male	Female	Male	Female	Male	Female	Male	Female
Native Fars	2.95	1.32	0.44	1.03	0.44	0.29	0.44	0.44
Turkmen	2.90	4.36	2.90	5.83	5.83	0	0	4.36
Sistani	1.89	3.79	1.26	0.63	3.15	0.63	1.89	1.89

ASD, Atrial septal defect; VSD, Ventricular septal defect; PDA, Patent ductus arteriosus

## Discussion

This study was conducted to explore the incidence rate and pattern of CHD for native Fars, Turkmen, and Sistani infants living in Gorgan, northern Iran. According to our results, the Turkmen newborns (15.93 per 1000) had the highest overall incidence rate of CHD, followed by the Sistani (12.27 per 1000) and native Fars (5.73per 1000) infants.

Several studies have reported racial/ethnic differences in the prevalence of CHD.^9^^, ^^10^^, ^^12^^, ^^13^^, ^^21^ The Sadig et al. study^9^ (1995) showed that the estimated prevalence of congenital heart defects requiring hospital admission was higher in Asian infants than in non-Asian ones (9.45 per 1000 vs. 4 56 per 1000; p value < 0.0001). Canfield et al.^10^ (2006) reported that, compared to the infants of non-Hispanic white mothers, the infants of non-Hispanic black mothers had a significantly high birth prevalence of Tetralogy of Fallot. In a population-based study, Correa-Villasenor et al.^21^ (1991) demonstrated that the white/black variation in cardiovascular malformations was found for several diagnostic groups of CHD. Indeed, Nembhard et al.^13^ (2009) reported that the Hispanic infants had the highest overall CHD prevalence (80.09 per 10 000 live births), followed by the non-Hispanic-white (79.11 per 10000 live births) and non-Hispanic black infants (77.67 per 10000 live births). Furthermore, Botto et al.^12^ (2001) showed that the incidence rate of CHD in the USA from 1968 through 1997 was 59.35 and 66.51 per 10000 in their white and black infants, respectively (RR = 1.12, 95%CI: 1.06-1.18). 

Differences in genetic predisposition, environmental exposures, access to care (diagnosis of CHDs), and response to physiologically-based CHD interventions may be the causes of racial/ethnic differences in the prevalence of CHD.^8^^, ^^15^^, ^^16^ Interpretation in terms of genetic and environmental causation, however, is not straightforward. For example, racial and ethnic variations are often associated with socioeconomic differences, lifestyle variation, cultural factors, and other factors that indicate environmental exposures according to the Botto et al. study^12^ in 2001.

In the present study, ASD, followed by ASD with VSD, was the most common lesion in the native Fars and Sistani ethnic groups, whereas in the Turkmen ethnic group, VSD with ASD, followed by ASD, was the most frequent lesion.

According to the Boto study^12^ (2001), a high overall occurrence rate for the blacks by comparison with the whites was primarily driven by a higher occurrence of peripheral pulmonic stenosis and ASD. However, transposition of the great arteries, truncus arteriosus, coarctation of the aorta, and aortic stenosis occurred more often among the whites. Also, racial differences were seen also within some broad defect categories. For example, transposition of the great arteries, but not tetralogy of Fallot, was more common among the whites. Similarly, among the left-side obstructive defects, coarctation of the aorta, but not hypoplastic left heart, was more common among the whites.

Nembhard^13^ (2010) drew upon data from the Florida Birth Defects Registry (FBDR) during 1998-2003 and reported that compared to the non-Hispanic whites, the non-Hispanic blacks had high rates of pulmonary valve atresia/stenosis but lower frequencies of aortic valve atresia/stenosis and VSD. The Hispanics had lower rates of aortic valve atresia/stenosis and atrioventricular septal defects than the non-Hispanic whites. Elsewhere, the Sadig et al. study^9^ (1995) showed that the Asian infants had a higher proportion of complex CHD (7% vs. 2.1%; p value < 0.001), whereas coarctation of the aorta was more common in the non-Asians (3% vs. 9.1%; p value = 0.003). Also, the proportion of admissions for persistent arterial duct seemed to be significantly higher in the Asian infants than in the non-Asian infants. Furthermore, a study carried out in Chinese people reported that in the live births, the top three lesions were VSD, PDA, and ASD, which accounted for 34.0%, 23.7%, and 10.8% of all lesions, respectively.^22^ Also, the WU et al. study^23^ (2010) in Taiwan demonstrated that VSD was the most common defect, followed by secundum ASD. In addition, female predominance was observed in VSD, ASD II, PDA, and endocardial cushion defect, while male predominance was seen in transposition of the great arteries, tetralogy of Fallot, PDA, and pulmonary stenosis.

The Fixler study^14^ reported that the white children had higher prevalence rates for aortic stenosis, endocardial cushion defect, and VSD. In that study, the Mexican-American children had the lowest rate for hypoplastic left heart syndrome.

In our study, the incidence rates of ASD and VSD in the native Fars group and VSD in Turkmen and Sistani groups were more common in the males than in the females, whereas ASD with VSD in the native Fars and Turkmen groups, and ASD in the Sistani subjects were more common in the females than in the males.

The Nembhard study^8^ (2010) by using data from the Florida Birth Defects Registry (FBDR) during 1998–2003 reported that compared with the non-Hispanic whites, the non-Hispanic black males had significantly increased rates of pulmonary valve atresia/ stenosis but lower prevalence of aortic valve atresia/stenosis and VSD. Also, the Hispanic males had lower rates of aortic valve atresia/stenosis, coarctation of the aorta, and VSD. On the other hand, the non-Hispanic black females had significantly lower rates of VSD and the Hispanic females had lower rates of Tetralogy of Fallot, VSD, and atrioventricular septal defect than the non-Hispanic whites.

According to the Mc Bride et al. study^20^ (2005), the prevalence ratios were significantly higher for the male sex. Also, the racial difference of higher rates for the whites compared to the blacks was confirmed for coarctation of the aorta and obstruction of the left ventricular outflow tract malformations. However, the rate differences between the white and black groups were not significant for the aortic valve stenosis and hypoplastic left heart syndrome cases.

Female predominance was reported in VSD, ASD, PDA, and endocardial cushion defect, whereas male predominance was observed in the transposition of the great arteries, Tetralogy of Fallot, PDA, and pulmonary stenosis.^23^

Storch and Mannick^19^ (1992) reported that the prevalence of atrioventricular canal defect per 1000 live births was significantly higher for the black females than that for the black males and for the white females compared to the white males. Also, complete transposition of the great arteries was significantly higher for the white males than for the white females. In contrast, transposition of the great arteries was not significantly different between the black males and the black females. Additionally, they demonstrated that obstructive left heart syndrome aortic stenosis and/or coarctation of the aorta was significantly higher for the white males than that for the white females.

The explanations for differences in the sex ratios by ethnicity are not readily clear. The Shaw et al. study^24^ reported that many defects have deviations in the ratio of the affected males versus the affected females and Gorlin^25^ found differences in the sex ratios by ethnicity for infants with cleft lips; nonetheless, no explanations for these deviations have been forthcoming. It is possible that the racial/ethnic differences can be the result of the differential loss of CHD-affected pregnancies during the gestational period rather than true differences in the incidence of CHD. As our study was conducted only on live births, we did not report spontaneous miscarriages, fetal deaths, and elective terminations. Needless to say, incidence can be affected by the aforementioned items.^26^

It is also possible that differences in the sex ratios by ethnicity might be due to racial/ethnic and sex-specific differences in the diagnosis of CHD after birth^8^ or might be in consequence of having access to pediatric care and treatment.

First and foremost among the limitations of the current study is that the true incidence of CHD within a population can only be determined with complete ascertainment of all fetal outcomes and we were not able to completely establish all fetal outcomes, primarily because spontaneous and induced abortions before 20 weeks’ gestation were not documented in this study. And nor were we able to state the number of severely ill children who died during the initial steps of resuscitation (before echocardiography could be performed). Indeed, we only had access to the gender and ethnicity in the healthy newborns (without malformations); therefore, we could not adjust the effect of such potential confounders as the mother’ age and parity in this study.

## Conclusion

For the first time, we found racial/ethnic and sex-specific differences in the incidence and pattern of CHD in Gorgan, northern Iran. Our findings showed a significant difference in CHD between the native Fars, Turkmen, and Sistani infants. While ASD was more common among the native Fars and Sistani infants, ASD with VSD was more frequent in the Turkmen children.

These findings should help establish a database for future studies focusing on the etiology and ethnic disparity of CHD in the region. The findings could lead to valuable changes in health policies for the improvement of diagnostic and therapeutic facilities.

CHD needs regular monitoring so as to permit optimal growth and development. Early diagnosis and timely intervention will, therefore, reduce the morbidity and mortality to a large extent.

## Notes

***This paper should be cited as:*** Nikyar B, Sedehi M, Qorbani M, Nikyar A, Golalipour MJ. Ethnical Variations in the Incidence of Congenital Heart Defects in Gorgan, Northern Iran: A Single-Center Study. J Teh Univ Heart Ctr 2014;9(1):9-14.
